# Correlation between free-field cortical auditory evoked potentials and behavioral audiometry thresholds in pediatric patients with hearing aids or cochlear implants

**DOI:** 10.1016/j.bjorl.2026.101922

**Published:** 2026-11-04

**Authors:** Yang Ruan, Yuan Wang, Wendi Shi, Fangjun Zhang

**Affiliations:** a Northwest University First Hospital, P.R. China; b Hangzhou Hui’er Hearing Instrument & Technique Co. Ltd., P.R. China; c Hui’er Artificial Intelligence Hearing Research Center, Zhejiang Province, China

**Keywords:** Cortical Auditory Evoked Potentials (CAEPs), Behavioral audiometry, Correlation, Hearing aids, Cochlear implant, Clinical application

## Abstract

**Objectives:**

To determine the correlation between hearing thresholds obtained using free-field Cortical Auditory Evoked Potentials (CAEPs) and behavioral audiometry in children with sensorineural hearing loss after hearing-aids or cochlear-implant intervention, and to determine whether P1 latency and N1-P1 inter-peak amplitude can be used to assess hearing device outcome in young children.

**Methods:**

Thirty-three children (2–14 years) with mild-to-profound sensorineural hearing loss were divided into a cochlear-implant group (22-ears) and a hearing-aid group (27-ears). Tone burst stimuli at 0.5-, 1-, 2-, and 4-kHz were used to evoke CAEPs and behavioral audiometry thresholds in a free field. The CAEP P1 latency and N1-P1 inter-peak amplitude were recorded.

**Results:**

Free-field tone-burst CAEP P1 response thresholds were positively correlated with free-field behavioral audiometry thresholds at 0.5-, 1-, 2-, and 4-kHz in both the hearing-aid group (Pearson correlation coefficients: 0.567, 0.670, 0.637, and 0.762, respectively, p < 0.01) and the cochlear-implant group (0.825, 0.662, 0.538, and 0.567 respectively). At the tested frequencies, the mean differences between the 2 testing methods were 5–8 dB in the cochlear-implants group and 1–6 dB in the hearing-aid group. In both groups, the CAEP P1 latencies were 130–150 ms, and the N1-P1 inter-peak amplitudes were 6 μV.

**Conclusions:**

When evaluating the efficiency of hearing interventions in clinical settings, free-field CAEP testing using tone-burst stimuli may serve as a clinically useful supplementary tool for pediatric patients who have difficulty cooperating with behavioral audiometry, provided its limitations are taken into account.

**Level of evidence:**

This study is a diagnostic study providing Level 2b evidence. The results demonstrate a significant correlation between CAEP and behavioral hearing thresholds in children using hearing devices, supporting its potential applicable value as an objective clinical assessment tool.

## Highlights

• Technological innovation and methodological clinical adapt.
• Directly addressing clinical problems through targeted subject selection.
• Direct clinical implications and translation potential.

## Introduction

In 2021, 34 million children worldwide^1^ and 0.17% of children aged 0–14 in China^2^ were estimated to have hearing impairment. Depending on the developmental stage, hearing loss can adversely affect auditory cognition, speech development, social adaptation, and communication abilities,^3^^,^^4^ necessitating appropriate and individualized management. The under-amplification of hearing devices is a common cause of delayed language acquisition and auditory system development in hearing-impaired children. Clinical interventions require accurate determination of hearing thresholds, a challenging task in pediatric patients. In our experience, hearing thresholds obtained using subjective methods, such as behavioral audiometry with speech stimuli, are less satisfactory in pediatric patients, due to their poor cooperation, young age, and etiology of hearing loss. Currently, the protocols for pediatric amplification in the UK, Australia, and China^5^ recommend objective measures for early hearing screening and diagnosis in pediatric patients.

Objective audiological electrophysiological tests include the Auditory Brainstem Response (ABR) test and the Cortical Auditory Evoked Potential (CAEP) test.^6^ With the hearing thresholds estimated using electrophysiological tests, audiologists can decide hearing interventions such as cochlear implantation or hearing-aids, although some uncertainty exists regarding the adequate amplification. Therefore, audiologists tend to be conservative in their prescribed amplification when applying electrophysiological results for hearing-aid fitting^3^ in pediatric patients. The frequency-specific free-field chirp ABR test has been widely used clinically to provide a reference for audibility after hearing-aid fitting and cochlear-implant mapping. However, ABRs are generated at the level of the brainstem, with minimal information about the speech cognition at cortical level of the patient. ABR testing is significantly affected by patients’ sleep states. When a child is unable to fall into deep sleep, the ABR test becomes difficult to perform. Moreover, ABRs originate at the brainstem level and cannot adequately reflect the development of a child’s cognitive speech and language abilities. Therefore, there is an urgent clinical need for a fast, accurate, and objective assessment method that can evaluate the amplification effect of hearing devices in awake children and monitor development up to the cortical level.

Compared with ABR testing, CAEP testing offers key advantages for pediatric assessment: (1) It reflects cortical sound processing and speech perception capability; (2) It is performed in awake children, avoiding unnecessary sedation; (3) It uses longer stimuli that better simulate real-world sound environment; and (4) it provides more reliable threshold estimation in conditions like auditory neuropathy. These features make CAEP testing a promising complementary tool to ABR testing for verifying hearing devices’ performance and monitoring cortical auditory maturation. The CAEP is an obligatory neural response that originates from various auditory generators in the temporal lobe, and reflects the sound-recognition ability of the auditory cortex. The CAEP comprises P1, N1, and P2 components, whose latencies lie between 50 and 500 ms.^7^ The P1 wave is the most prominent in the pediatric population, and can be detected at approximately 300 ms in newborns;^8^ its latency then shortens and stabilizes between 50 and 80 ms in adulthood. Thus, P1 latency serves as a biological marker of auditory-system maturity.^9^ Unlike most electrophysiological tests, CAEP testing is performed while the subject is alert and awake. Thus, CAEP testing with frequency-specific stimuli has the potential of providing insights into aided audibility.

This study aims to measure the degree of correlation between auditory thresholds obtained using the objective CAEP test (especially P1 latency) and those obtained using subjective behavioral audiometry in children with sensorineural hearing loss who have undergone cochlear implantation or hearing-aids fitting. A strong correlation between the 2 methods would indicate that CAEP testing is a feasible alternative for assessing hearing-device efficiency in pediatric patients.

## Methods

### Research subjects

This study was approved by the ethics committee of International Education College, Zhejiang Chinese Medical University (20230118-3). The study procedures were fully explained, and written informed consent was obtained from all patients and/or their legal guardians. The patients/guardians consented to the publication of anonymous data, and the study complies with the requirements of privacy protection in medical ethical standards.

We prospectively enrolled children with mild-to-profound sensorineural hearing loss who visited the Hui’er International Hearing Center between 20/01/2023 and 20/11/2023. Pediatric subjects were eligible for this study provided they met the following inclusion criteria: (1) Mild-to-profound sensorineural hearing impairment bilaterally; (2) Cooperate well during behavioral audiometry with high repeatability; (3) Regular hearing-aids or cochlear-implant use for more than 1-year, with daily usage over 10 h; (4) Normal middle ear function (type A tympanogram); (5) No clinical diagnosis of post-cochlear nerve dysfunction (such as auditory neuropathy, small auditory nerve, and cochlear hypoplasia); (6) No psychological or mental disorders (such as autism or attention-deficit/hyperactivity disorder); (7) Completion of more than 1 semester of speech rehabilitation; (8) Cochlear implant subjects meeting the surgical indications and having undergone successful implantation according to the Cochlear Implantation Work Guide (2013)^10^; (9) Duration between cochlear-implant activation and recruitment exceeding 6-months.

The following exclusion criteria were applied: (1) Children with non-permanent or permanent conductive hearing loss; (2) Children diagnosed with auditory neuropathy spectrum disorder; (3) Children with unilateral hearing loss; (4) Children with cognitive, behavioral, or developmental disorders (e.g., autism spectrum disorder, intellectual disability, attention-deficit/hyperactivity disorder) that would preclude reliable behavioral hearing assessment. (5) Children with neurological disorders or abnormalities in brain imaging that may affect the central auditory pathway.

### Pre-experiment preparation

Before the hearing-threshold evaluations, otoscopy was performed in all children to examine the ear canal and tympanic membrane, and exclude any obstruction or abnormalities. Next, tympanometry was performed for all children using a Titan Suite device (Interacoustics, Beijing, China). Any patient with abnormal compliance, middle-ear pressure, ear-canal volume, or otoscopic findings was excluded from the study.

In children with hearing aids, Verifit2.0 (Audioscan) was used for real-ear measurement of the hearing aid to ensure that children were in the optimal fitted state.^11^ Ear molds were checked to ensure there were no cracks that might cause sound leakage or feedback. Special features of the hearing aids, including directional microphones, frequency shifting, and noise reduction, were set at basic.

In children with cochlear implants, the electrically evoked ABR and electrically evoked stapedial reflex threshold^12^, ^13^, ^14^ were assessed. The T- and C-values of the cochlear implant were set as a reference for programming settings. The functions of the external unit and the implant were checked according to the manufacturer’s manual to ensure optimal aiding performance.

### Experimental set-up

All tests were conducted in an electrophysiology testing room, with background noise <35 dB Sound Pressure Level (SPL). Prior to testing, the instruments and environment were calibrated. The sound field environment was aligned with the free-field calibration standard GB/T16296-1996. Only monaural listening was used during the experiment. During testing, the hearing device in the contralateral ear (if any) was turned off, and the external cochlear component was removed. Earplugs were used for masking if necessary. All tests were conducted by the same 2 audiologists, each of whom has 5-years of experience.

### Experimental procedure

#### Free-field behavioral audiometry

Free-field audiometry methods (conditioned play or the conventional hand-raising method) were chosen according to the age and understanding capabilities of the child. The Interacoustics AC40 audiometer was used. The sound speaker was placed 1 meter away in the horizontal direction from the microphone of the hearing device. After free-field calibration, frequency-specific warbles were used to perform conditioned training on the tested subject. After obtaining accurate repeatable responses, the Hughson-Westlake method was used to obtain aided thresholds at 0.5-, 1-, 2-, and 4-kHz.

#### Free-field CAEP testing

The child was in a relaxed sitting position, accompanied by a guardian, and remained awake during the test. The child was given the option of a book or silent cartoon clips for entertainment. The speakers were placed 1 meter away in the horizontal direction from the subject’s ears, in alignment with the microphone of the hearing device. Nuprep gel was used to exfoliate the electrode placement areas, removing sebum and debris. Electrodes were placed on the vertex, mastoid process, and forehead between the eyebrows. The Neuro-Audio electrophysiological test platform was chosen for this experiment. The following settings were used while observing P1 wave emergence: electrode impedance and inter-electrode resistance, <3 kΩ; band-pass filter, 0.3–30 Hz; stimulation rate, 1-time/s; sampling rate, 5000 Hz; time window, 500 ms; artifact rejection, ±100 μV; and average number of repetitions, >50. The waveforms at each recording site were averaged using a computer to ensure test repeatability. A typical qualitative example of free-field CAEP testing is shown in Fig. 1. Tone bursts of 0.5-, 1-, 2-, and 4-kHz were used to evoke the P1 wave.^15^ The audiologist monitored the subject’s status throughout the testing procedure, allowing breaks in between to maintain the accuracy of CAEP testing. CAEP thresholds were determined using a modified Hughson-Westlake descending method, starting at 20 dB SL. All P1 latencies were recorded at the individual threshold intensity for each frequency in each ear (i.e., 0 dB sensation level relative to the CAEP threshold). A representative example of the free-field CAEP response waveform recorded at each frequency is shown in Fig. 1. A representative example of the relationship between free-field tone-burst CAEP P1 thresholds and free-field behavioral audiometry thresholds at 0.5-, 1-, 2-, and 4-kHz in children with hearing aids and children with cochlear implants is shown in Supplemental Fig. S1 and Supplemental Fig. S2.

**Fig. 1:**
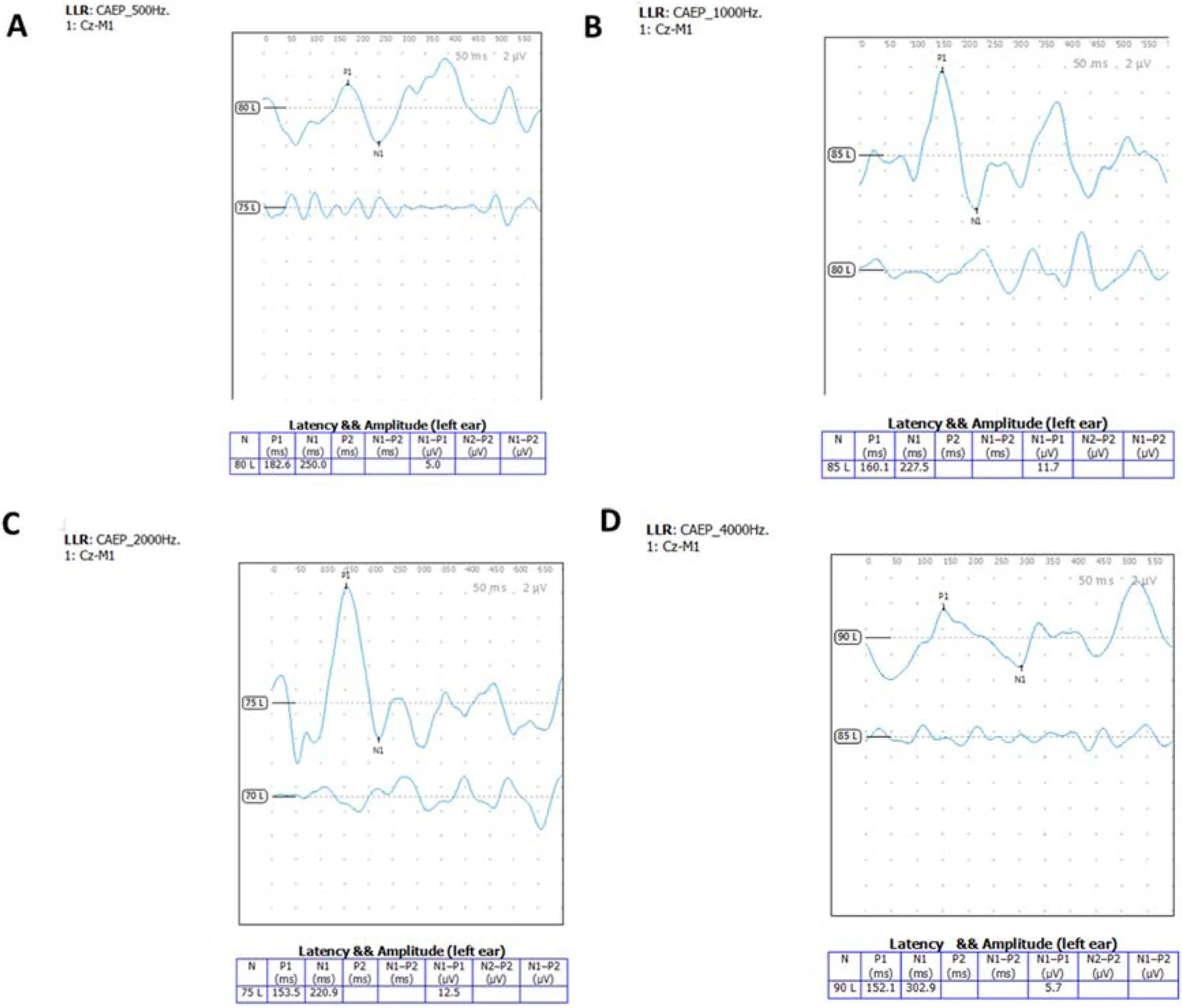
Schematic diagram of free-field CAEP testing of hearing-impaired children after auditory intervention tested in a free field at 0.5 (A), 1 (B), 2 (C), and 4 (D) kHz. CAEP, Cortical Auditory Evoked Potential.

### Statistical analysis

The normality of data distribution was assessed using the Shapiro-Wilk test. Although some frequency-specific datasets showed slight deviations from normality, mean and standard deviation were reported to maintain consistency with the published literature and facilitate cross-study comparison. Non-parametric tests (Spearman rank correlation) yielded similar significance levels, supporting the robustness of our findings.

The results of free-field behavioral audiometry and free-field tone-burst CAEP P1 thresholds at each test frequency of 0.5-, 1-, 2-, and 4-kHz were collected, and analyzed using SPSS v.26.0 (Fig. 2). The differences between subjective and objective test thresholds were expressed as the mean ± standard deviation. Pearson correlation analysis was used to test the correlation between the subjective and objective free-field test results at the corresponding test frequencies in each group. An *r* coefficient >0.5 and p < 0.01 were considered to indicate a strong correlation and a statistically significant difference.

**Fig. 2:**
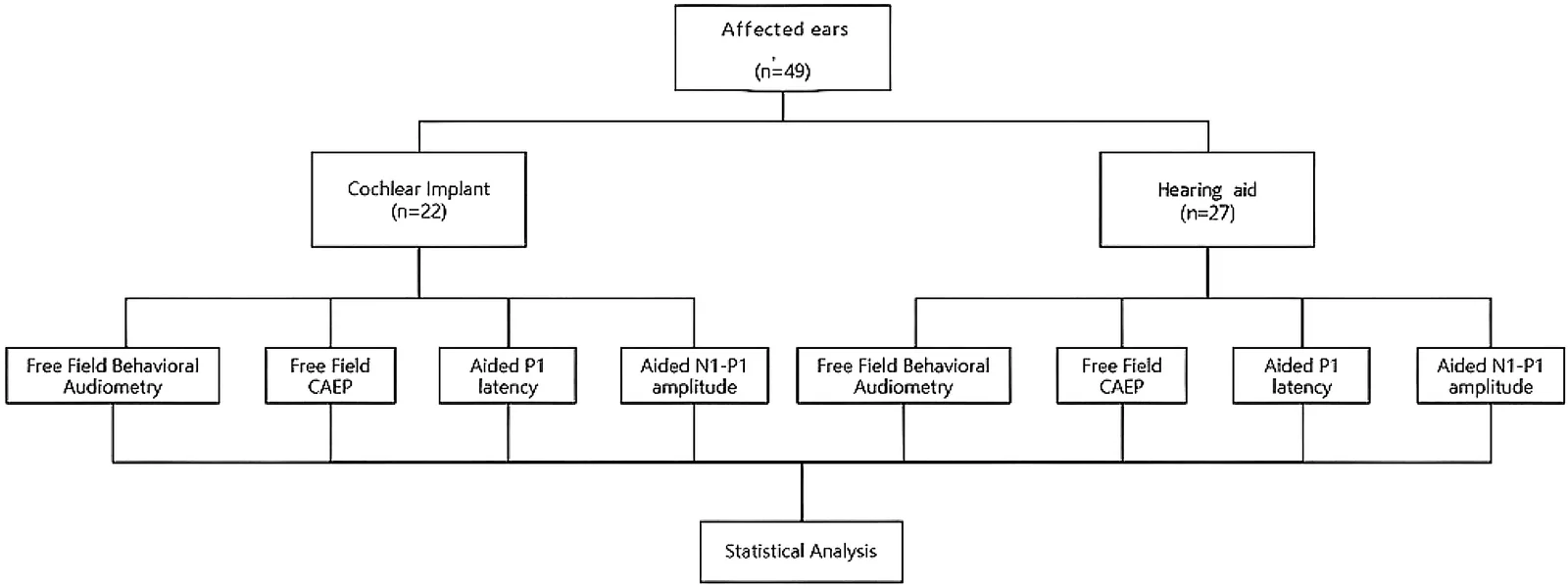
Technical flow chart on the correlation between free-field tone burst CAEP and free-field behavioral audiometry. CAEP, Cortical Auditory Evoked Potential.

## Results

### General information

This study enrolled 33 children with sensorineural hearing loss (49 affected ears). A total of 17-ears did not meet the inclusion criteria for the following reasons: they had mixed or conductive hearing loss, thereby not meeting the strict criteria for isolated sensorineural hearing loss; they were suspected of having retrocochlear lesions (e.g., auditory neuropathy) involving the auditory nerve or central auditory pathways; they had confounding factors such as a history of external or middle ear disease, or a diagnosis of syndromic hearing loss associated with other known syndromes; or they had not met the intervention criteria (e.g., less than 1 year hearing aid intervention or less than 6-month cochlear implant usage).

The patients included 19 girls and 14 boys, with ages ranging from 2- to 14-years (mean: 5.32 ± 2.73 years). Of the 49 affected ears, 22 had received a cochlear implant, and 27 had been fitted with a hearing aid. The average hearing loss in the overall cohort was 73.85 ± 20.48 dB Hearing Level (HL). The mean age at hearing intervention did not differ between the cochlear implant group (4.86 ± 2.35 years) and the hearing aid group (5.56 ± 2.96 years, p = 0.36). However, the mean duration of hearing intervention was significantly shorter in the cochlear implant group (1.05 ± 0.29 years) than in the hearing aid group (3.30 ± 2.52 years, p < 0.001).

The relatively large standard deviations observed in Table 1, Table 2, Table 3, Table 4, Table 5 indicate considerable inter-subject variability. Potential contributors include the wide age range (2–14 years) of the participants, differences in hearing device type and duration of use, and the analytical approach of treating each ear as an independent unit (both ears were studied in only 4 children, and none had symmetrical device types, i.e., cochlear implant in one ear and hearing aid in the contralateral ear). A sensitivity analysis excluding the 4 children with bilateral data did not materially change the correlation coefficients (data not shown). These factors are further addressed in the Discussion.

**Table 1:** Free-field aided behavioral audiometry thresholds and CAEP P1 response thresholds in children with hearing aids.

Frequency (kHz)	0.5	1	2	4
Behavioral audiometry threshold (dB HL)	36.67 ± 13.37	39.81 ± 13.76	40.37 ± 11.68	42.04 ± 13.18
CAEP P1 response threshold (dB HL)	44.26 ± 15.97	43.33 ± 11.18	42.78 ± 9.34	43.70 ± 10.80
Difference (dB HL)	6.30 ± 8.27	3.52 ± 8.86	2.41 ± 9.84	1.67 ± 10.83

CAEP, Cortical Auditory Evoked Potential; HL, Hearing Level.

**Table 2:** CAEP P1 response threshold latency and N1-P1 inter-peak amplitude in children with hearing aids (measured at individual threshold intensity, 0 dB SL).

Frequency (kHz)	0.5	1	2	4
P1 latency (ms)	146.52 ± 24.47	139.67 ± 20.66	151.94 ± 34.35	142.73 ± 30.96
N1-P1 inter-peak amplitude (μV)	5.92 ± 2.09	6.37 ± 2.35	6.25 ± 2.57	6.55 ± 2.59

CAEP, Cortical Auditory Evoked Potential.

**Table 3:** Correlation between free-field aided behavioral audiometry thresholds and CAEP P1 response thresholds in children using hearing aids.

Frequency (kHz)	0.5	1	2	4
Pearson’s correlation coefficient (r)	0.83^a^	0.66^a^	0.54	0.57
p-value	<0.001	<0.001	0.001	0.002

CAEP, Cortical Auditory Evoked Potential.

a p < 0.001.

**Table 4:** Free-field aided behavioral audiometry thresholds and CAEP P1 response thresholds in children with cochlear implants.

Frequency (kHz)	0.5	1	2	4
Behavioral audiometry threshold (dB HL)	38.64 ± 7.59	36.14 ± 7.55	37.50 ± 8.42	39.32 ± 6.95
CAEP P1 response threshold (dB HL)	46.82 ± 9.45	44.09 ± 6.84	44.09 ± 10.87	44.55 ± 8.44
Difference (dB HL)	8.18 ± 8.09	7.95 ± 5.70	6.59 ± 7.13	5.22 ± 5.22

CAEP, Cortical Auditory Evoked Potential; HL, Hearing Level.

**Table 5:** CAEP P1 response threshold latency and N1-P1 inter-peak amplitude in the cochlear-implant group (measured at individual threshold intensity, 0 dB SL).

Frequency (kHz)	0.5	1	2	4
P1 latency (ms)	147.26 ± 42.32	143.05 ± 40.22	144.20 ± 41.67	130.13 ± 33.51
N1-P1 inter-peak amplitude (μV)	5.95 ± 2.73	6.41 ± 2.72	5.61 ± 1.67	6.63 ± 2.17

CAEP, Cortical Auditory Evoked Potential.

### Hearing-aid group

The differences between the free-field CAEP and behavioral audiometry thresholds at each test frequency were between 2 and 6 dB among children using hearing aids (Table 1).

The free-field CAEP P1 response threshold and free-field behavioral audiometry threshold were significantly correlated at each tested frequency (p < 0.01, Pearson correlation analysis). The correlation coefficients indicated a high correlation at 0.5 kHz and moderate correlations at 1 kHz, 2 kHz, and 4 kHz. The correlation was highest at low frequencies and decreased toward high frequencies (Table 3).

The P1 wave threshold latency was 139–151 ms, and the N1-P1 inter-wave amplitude was approximately 6 μV in the hearing-aid group (Table 2). All reported latencies were recorded at the individual CAEP threshold intensity for each frequency (0 dB SL); therefore, pooling latencies across ears with different CAEP response thresholds is methodologically valid, as they represent the same relative sensation level.

### Cochlear-implant group

The differences between the free-field tone-burst CAEP P1 response thresholds and free-field behavioral audiometry thresholds in children with cochlear implants were in the range of 5–8 dB HL (Table 4), with the differences narrowed from low frequencies to high frequencies.

The results of both test methods showed significant linear correlation across testing frequencies in children with cochlear implants (p < 0.01, Table 5). The correlation coefficients revealed moderate correlations at 0.5 kHz, 1 kHz, and 2 kHz, and a high correlation at 4 kHz (Table 6).

**Table 6:** Correlation between free-field aided behavioral audiometry thresholds and CAEP P1 response thresholds in the cochlear-implant group.

Frequency (kHz)	0.5	1	2	4
Pearson’s correlation coefficient (*r*)	0.57	0.67	0.64	0.76^a^
p-value	0.006	0.001	0.002	<0.001

CAEP, Cortical Auditory Evoked Potential.

a p < 0.001.

The average free-field P1 response threshold latency was 130–147 ms, and the amplitude between the N1 and P1 waves was 6–7 μV in the cochlear-implant group (Table 5). As in the hearing-aid group, these latencies were recorded at the individual CAEP threshold intensity (0 dB SL).

## Discussion

Free-field behavioral audiometry, free-field ABR, free-field CAEP, and various assessments have been widely used for hearing device verification.^16^ Adults with normal cognition have a deviation of ±5 dB during pure-tone audiometry testing.^15^ This variability is larger in children. Speech test stimuli are often used during audiological testing; however, their use has limitations in certain situations, such as pediatric patients and patients speak dialect, which makes accurate evaluation of hearing-device performance difficult.^17^^,^^18^ Hearing-assessment questionnaires such as LittlEARS, PEACH, and IT-MAIS are also widely used to evaluate children’s responses to sounds and speech. However, these methods are limited due to variability and bias from parents and observation.

CAEP is a long-latency evoked potential in response to sound stimuli, and has the potential to reflect the integrity of the auditory pathway, while providing insights into hearing recognition and threshold prediction.^19^^,^^20^ The P1 and N1 components emerge during the neonatal period. With auditory system maturation, P1-N1 amplitude diminishes, and P1 latency shortens. Therefore, P1-N1 amplitude and P1 latency have scientific value as biomarkers of auditory system maturity. P1 wave latency is approximately 100 ms in children aged 2–12 years, and eventually stabilizes at 50–80 ms in adulthood.^21^ The general shortening in this CAEP latency is in phase with the neural myelination changes in the ascending auditory pathway. While free-field CAEP is often used for auditory-pathway integrity assessment before cochlear implantation, CAEP testing might also be feasible for post-intervention cochlear-implant and hearing-aid verification.^22^, ^23^, ^24^

The advantages of CAEP testing for hearing-device verification include the following: (1) CAEP reflects the auditory integrity of the auditory pathway better than short-latency evoked potentials like ABRs or auditory steady-state responses. (2) The shortening of P1 latency and decrease in P1 amplitude are highly correlated to the development and maturation of the auditory cortex. P1 latency is greatly affected by auditory experience and highly correlated with speech-perception ability. In the same age group, children with longer P1 latency often demonstrate poor auditory and speech abilities, while children with shorter P1 latency have better speech-perception ability.^9^^,^^12^^,^^25^ (3) CAEP testing can be performed in waking subjects. (4) The long duration of sound stimulation used for CAEP testing can fully activate the compression algorithm of the hearing device, mimicking environmental sounds in daily life.^26^, ^27^, ^28^ (5) Short latency electrophysiological responses such as ABR depend on neural synchronization of the auditory nerve. CAEP can be an advantage when testing patients with auditory neuropathy spectrum disorder, as the CAEP response threshold better correlates with the actual hearing threshold in patients with this disorder,^27^ who have absent ABRs even with mild audiometric hearing loss. Therefore, CAEP has an important reference value for evaluating the developmental status of the auditory center or the auditory neural plasticity of children, especially in children who are unable to cooperate with subjective testing.^9^ However, in clinical practice, the application of CAEP testing is also influenced by factors such as age, stimulus parameters, and subject state. The differences between CAEP threshold and behavioral audiometry thresholds vary considerably across studies. Durante et al. found that the difference between CAEP thresholds and behavioral audiometry thresholds was 14.5 dB in normal-hearing individuals and 7.8 dB in individuals with hearing loss.^29^

Many studies have reported the application of free-field CAEP to evaluate the efficiency of hearing-device intervention.^30^, ^31^, ^32^, ^33^ Several authors have confirmed a strong correlation between CAEP and free-field behavioral audiometry thresholds obtained using frequency-specific tone-burst stimuli or the speech tokens /m/, /g/, and /t/ in children with sensorineural hearing loss after hearing intervention.^19^^,^^22^^,^^28^^,^^32^^,^^34^, ^35^, ^36^, ^37^ Several studies have found that CAEP correlates with audibility, scale assessment, and speech recognition.^38^ Van Dun et al. and Gardner-Berry et al. found that audibility after hearing-aid fitting was significantly correlated with CAEP elicitation, and least correlated with the cause and degree of hearing loss.^35^^,^^39^ Xie et al. have discussed the effect of CAEP as an adjustable response after cochlear implantation, and concluded that CAEP is more strongly correlated with behavioral audiometry thresholds than with electrophysiological examination results such as ABR.^9^

CAEP elicitation is largely relevant to auditory experience. Different sound stimuli can induce CAEP responses.^40^ Wang et al. used frequency-specific tone bursts to monitor the development of auditory neural pathways after cochlear implantation in prelingually impaired children.^41^ Loudness balance in bimodal listening can avoid the lateralization of the auditory cerebral cortex after cochlear implantation and prevent auditory deprivation of the non-implanted ear.^42^ Liu et al. compared the characteristics of the CAEP P1 wave in children with bilateral hearing aids and monaural cochlear implants; they concluded that the P1 wave amplitude increases, and P1 latency tends to shorten after monaural cochlear implantation.^20^ Yan et al. reported that loudness balance reflects only a portion of the intervention effect in the setting of bimodal intervention; interestingly, when loudness balance is restored, CAEP P1 latency shortens and P1 amplitude increases, signifying a faster auditory modulation with bimodal hearing.^12^

To our knowledge, no study has examined the correlation between free-field CAEP and behavioral audiometry with short tone bursts as test stimuli in children who have undergone auditory intervention. We used frequency-specific short tone bursts at 0.5-, 1-, 2-, and 4-kHz to compare free-field CAEP P1 response thresholds with free-field behavioral audiometry thresholds after cochlear implantation and hearing-aid fitting. We found that after both intervention methods, the free-field CAEP P1 response thresholds were significantly correlated with the free-field behavioral audiometry thresholds (p < 0.01), indicating that CAEP testing can be used to clinically estimate children’s behavioral hearing thresholds. However, cross-validation between subjective and objective tests is necessary to better guide clinical hearing-aid fitting and cochlear-implant programming.

A moderate correlation (*r* = 0.54–0.67) was observed at several frequencies. While this is statistically significant, its clinical utility depends on the specific goal. For screening or gross estimation of audibility, a moderate correlation may be acceptable as a fallback when behavioral testing is impossible. However, for fine-tuning hearing-aid gain or cochlear-implant mapping at individual frequencies, a high correlation (e.g., *r* > 0.8) would be more desirable.

The average difference between the 2 thresholds was 5–8 dB in the cochlear-implant group and 2–6 dB in the hearing-aid group. P1 latency after cochlear implantation was 130–147 ms; the higher the frequency, the shorter the latency. In the hearing-aid group, P1 latency was 139–151 ms, demonstrating the strongest correlation at 4 kHz, and gradually decreasing towards the lower frequencies. In the cochlear-implant group, the correlation of P1 latency with the test frequencies was highest at the high frequencies, and gradually decreased towards the low frequencies. In the hearing-aid group, the correlation was highest at low frequencies, and decreased towards high frequencies. A possible reason for this phenomenon is that the electrodes of the cochlear implant are inserted from the basal turn of the cochlear round window, and spiral along the modiolus from the bottom to the apex. Most cochlear electrodes are situated at the basal turn rather than at the apex, and thus, have better high-frequency compensation than low-frequency compensation. The standard deviation of the CAEP P1 wave threshold latency in this study is relatively large, which may be related to the wide age range of the study participants as well as the use of each ear as an independent unit. Factors such as individual differences in auditory development levels, children’s cooperation, interpretation bias in P1 waveform identification, and variations in device calibration may all contribute to the differences. Although intervention with hearing aids or cochlear implants can compensate for residual hearing in children with hearing loss, the threshold latency of the P1 wave in CAEP testing remains prolonged compared with that in normal children. This may be associated with the maturity of the auditory cortex as well as children’s auditory and speech abilities. In terms of auditory cortex maturity, congenital hearing loss leads to long-term auditory deprivation, resulting in stagnation or retardation of central auditory system development. If the critical developmental period before approximately 3.5-years of age is missed, cortical developmental catch-up will be compromised even after hearing-device implantation.^43^ Additionally, cross-sensory cortical reorganization may occur,^44^ rendering P1 latency more closely correlated with auditory age (duration of device use) rather than chronological age. At the auditory and speech level, studies have found that prolonged P1 latency is negatively correlated with scores on scales such as the Categories of Auditory Performance and the Speech Intelligibility Rating,^45^ objectively reflecting difficulties in sound discrimination and speech comprehension. However, this does not equate to functional outcomes ‒ some children, despite having longer P1 latencies than their normal-hearing peers, can still achieve satisfactory academic performance within the normal range, suggesting the possible existence of compensatory processing pathways in the brain.^46^ In summary, delayed P1 latency reflects a “temporal gap” in auditory cortex development and experience-dependent plasticity. Early intervention and bimodal stimulation can facilitate its maturation. Zhang et al. analyzed the programming and settings of cochlear implants after initial activation in children, and concluded that with appropriate programming, the dynamic range of each frequency increases, with the largest variable range in the high frequencies.^47^

## Conclusion

Free-field behavioral audiometry and free-field tone-burst CAEP were significantly correlated with each other, and were highly relevant at the tested frequencies in children with cochlear implants or hearing aids. Free-field tone-burst CAEP is an alternative for hearing-device validation in children with sensorineural hearing loss. The difference between the 2 free-field evaluations was 5–8 dB after cochlear implantation and 1–6 dB after hearing-aid intervention. The P1 latency threshold was approximately 130–150 ms, and the N1-P1 inter-peak amplitude was 6 μV after hearing intervention.

P1 latency was prolonged in our subjects, possibly due to their congenital hearing loss. Thus, even with appropriate hearing intervention, auditory development might be insufficient and delayed when compared with normal-hearing children.

While our findings support the potential of free-field CAEP testing, the study is limited by its relatively small sample size and the wide age range of the participants, which may introduce variability related to cortical maturation. Therefore, the results should be interpreted as preliminary, and future studies with larger, age-stratified samples are needed to validate the clinical utility of CAEP testing for individual device programming.

## ORCID ID

Yang Ruan: 0009-0005-5537-0388

Yuan Wang: 0009-0008-2177-0422

Wendi Shi: 0009-0001-4169-5510

Fangjun Zhang: 0009-0002-7598-1029

## Consent for publication

We declare that this article is original and has not been published in any other journal.

## Ethical considerations

This study was approved by the ethics committee of Zhejiang University of Traditional Chinese Medicine (20230118-3).

## Funding

This work was supported by the Key Research and Development Program of Hangzhou (Grant No. 2025SZD1A12); the Key Project of Zhejiang Health Science and Technology Program (Grant No. WKJ-ZJ-26066); and the CDPF Hearing and Speech Program (No. 2026CDPFHS-01).

## Data availability statement

Data supporting the conclusions in this study are available from the corresponding author upon reasonable request.

## Declaration of competing interest

The authors declare no potential conflicts of interest with respect to the research, authorship, and/or publication of this article.

## References

[1] World Health Organization (2021).

[2] Long M., Zheng X., Bu X. (2021).

[3] Mehta K., Mahon M., Van Dun B., Marriage J., Vickers D. (2020). Clinicians’ views of using cortical auditory evoked potentials (CAEP) in the permanent childhood hearing impairment patient pathway. Int J Audiol.

[4] Long M., Zheng X.Y. (2021).

[5] Chinese Society of Otorhinolaryngology Head and Neck Surgery Audiology Group (2024). Clinical practice guideline for pediatric hearing aid fitting (2024). Chin J Otorhinolaryngol Head Neck Surg.

[6] Li Q. (2021). Interpretation of the 2019 American Academy of Audiology “Clinical Cochlear Implantation Clinical Practice Guide”: patient selection and indications. Lin Chuang Er Bi Yan Hou Tou Jing Wai Ke Za Zhi.

[7] Guo Q.Q., Chen X.Q., Mo L.Y., Meng C. (2016). The application of cortical auditory evoked potentials in the assessment of hearing aid fitting for hearing-impaired children. J Audiol Speech Pathol..

[8] LY L., Zhong Y., Liu G., Guo Q.Q., Fu X.X., Qi B.E. (2020). Study about reference values of cortical auditory evoked potentials P1-N1-P2 in the aged with normal hearing induced by speech stimulus. J Clin Otorhinolaryngol Head Neck Surg.

[9] Xie Y.F., Pan T., Wang Y. (2022). Optimization of electrically evoked cortical auditory potentials p!-n1-p2 test parameters in pediatric patients. Chin J Otol..

[10] Editorial Board of Chinese Journal of Otorhinolaryngology Head and Neck Surgery (2014). Guideline of cochlear implant (2013). Zhonghua Er Bi Yan Hou Tou Jing Wai Ke Za Zhi.

[11] Expert Group on Universal Newborn Hearing Screening of the National [institution not specified] (2018). Guideline for the early diagnostic evaluation and intervention of hearing loss in infants. Zhonghua Er Bi Yan Hou Tou Jing Wai Ke Za Zhi..

[12] Yan X.H., Zhou M.Y., Zhao Y., Shi W.D., Wang Y.H. (2023). Adjustment and objective evaluation in bimodal cochlear implant and hearing aid intervention in infants. Chin J Otol..

[13] Huang M.P., Yang L., Li J., Li B., Zhou Z. (2023). Application of objective test in mapping after chinese cochlear implanting. J Audiol Speech Pathol..

[14] Shi W., Cheng Y., Bao X.H., Zheng H.C. (2019). The role of ESRT and EABR in Cochlear Implant Programming. Chin Sci J Hear Speech Rehabil..

[15] Cortical ERA - British Society of Audiology[EB/OL]. [2023-10-13]. https://www.thebsa.org.uk/resources/cortical-era/.

[16] Li L.S., Ruan Z., Tang M., Zhang J.Q., Li Y. (2021). Correlation between aided chirp-ABR thresholds and behavioral audiometry in children with external and middle ear malformation. Chin J Otorhinolaryngol Head Neck Surg..

[17] Tang M., Zhang P.P., Deng H.X., Shen Y., Cheng L.X. (2020). Correlation between eabr threshold and behavioral audiometry current level threshold in cochlear implant recipients. Chin J Otorhinolaryngol Head Neck Surg..

[18] Kan C., Liu H., Fu X.X., Chen X.Q., Wang S. (2013). Cochlear implant performance evaluation with cortical auditory evoked potential in a group of post-lingually deaf adults. Chin J Otorhinolaryngol Head Neck Surg..

[19] Hassaan M.R. (2011). Aided evoked cortical potential: an objective validation tool for hearing aid benefit. Egypt J Ear Nose Throat Allied Sci.

[20] Liu Y.D., Zheng Q., Ni G.J., Geng J.Q., Liu H.H. (2020). Study on the feature of electroencephalogram children with cochlear implant and hearing aids (bi-mode). J Audiol Speech Pathol..

[21] Liu H.H., Ni X. (2015). Application of auditory evoked potentials perceptions in children. Chin J Otol..

[22] Guo Q.Q., Chen X.Q., Mo L.Y., Meng C. (2016). Application of cortical auditory evoked potentials in the assessment of hearing aid fitting for hearing impaired children. Chin Sci J Hear Speech Rehabil..

[23] Bauer P.W., Sharma A., Martin K., Dorman M. (2006). Central auditory development in children with bilateral cochlear implants. Arch Otolaryngol Head Neck Surg..

[24] Wang J.B., Liang M.J., Liu J.H., Chen Y.B., Zheng Y.Q. (2019). Value and assessment of P1 component in children with CIs. Chin Arch Otolaryngol Head Neck Surg..

[25] Ji F. (2017). Review of clinical applications of auditory evoked potentials. Chin J Otol..

[26] Mehta K. (2020).

[27] Rance G., Cone-Wesson B., Wunderlich J., Dowell R. (2002). Speech perception and cortical event related potentials in children with auditory neuropathy. Ear Hear..

[28] Cone B., Whitaker R. (2013). Dynamics of infant cortical auditory evoked potentials (CAEPs) for tone and speech tokens. Int J Pediatr Otorhinolaryngol..

[29] Durante A.S., Wieselberg M.B., Roque N. (2017). Assessment of hearing threshold in adults with hearing loss using an automated system of cortical auditory evoked potential detection. Braz J Otorhinolaryngol..

[30] Leite R.A., Magliaro F.C.L., Raimundo J.C., Bento R.F., Matas C.G. (2018). Monitoring auditory cortical plasticity in hearing aid users with long latency auditory evoked potentials: a longitudinal study. Clinics (Sao Paulo)..

[31] Baydan M., Batuk M.O., Sennaroglu G. (2019). Relationship between aided cortical auditory evoked responses and aided behavioral thresholds. Int J Pediatr Otorhinolaryngol..

[32] Ching T.Y., Zhang V.W., Hou S., Van Buynder P. (2016). Cortical auditory evoked potentials reveal changes in audibility with nonlinear frequency compression in hearing aids for children: clinical implications. Semin Hear..

[33] Carter L., Golding M., Dillon H., Seymour J. (2010). The detection of infant cortical auditory evoked potentials (CAEPs) using statistical and visual detection techniques. J Am Acad Audiol..

[34] Zhang Y.M., Wang S.J., Liu H. (2023). Application of cortical auditory evoked potential in hearing assessment. J Audiol Speech Disord..

[35] Van Dun B., Carter L., Dillon H. (2012). Sensitivity of cortical auditory evoked potential detection for hearing-impaired infants in response to short speech sounds. Audiol Res..

[36] Easwar V., Glista D., Purcell D.W., Scollie S.D. (2012). Hearing aid processing changes tone burst onset: effect on cortical auditory evoked potentials in individuals with normal audiometric thresholds. Am J Audiol..

[37] Hazzaa N., Hassaan D.M., Hassaan A. (2016). Evaluation of nonlinear frequency compression hearing aids using speech P1 cortical auditory evoked potential. Hear Balance Commun.

[38] Xu H.Y., Li Y., Zha S.H. (2024). Correlation between the mean threshold of cortical auditory evoked potential and the threshold of speech recognition threshold in patients with sensorineural hearing loss. Chin J Otorhinolaryngol Head Neck Surg..

[39] Gardner-Berry K., Chang H., Ching T.Y., Hou S. (2016). Detection rates of cortical auditory evoked potentials at different sensation levels in infants with sensory/neural hearing loss and auditory neuropathy spectrum disorder. Semin Hear..

[40] Martins K.V.C., Gil D. (2017). Cortical auditory evoked potentials with simple (Tone Burst) and complex (Speech) stimuli in children with cochlear implant. Int Arch Otorhinolaryngol..

[41] Wang X.L., Wang C.F., Zhang P., Zheng Y.X., Mao Z.Y. (2020). Central auditory development： evidence from caep measurements in the congenitally deaf children after cochlear implant. J Audiol Speech Pathol..

[42] Wang K.Z., Zhang P.P., Zhu S.F., Shen Y., Zhang J., Shi W.D. (2019). The antagonistic effect of contralateral hearing aids on the rehabilitation of auditory function in patients with cochlear implants. Chin Sci J Hear Speech Rehabil..

[43] Sharma A., Dorman M.F., Spahr A.J. (2002). A sensitive period for the development of the central auditory system in children with cochlear implants: implications for age of implantation. Ear Hear..

[44] Dorman M.F., Sharma A., Gilley P., Martin K., Roland P. (2007). Central auditory development: evidence from CAEP measurements in children fit with cochlear implants. J Commun Disord..

[45] Lu T., Zhao X., Zhu N. (2025). Correlation analysis between auditory cortical evoked potentials and auditory speech abilities in hearing-impaired children under bimodal hearing intervention. Chin J Ophthalmol Otorhinolaryngol..

[46] Roslle N., Wan Hashim W.F., Khairuddin K.F., Abdullah A., Ismail R., Hashim N.D. (2025). Comparison of cortical auditory maturation between children with cochlear implants VS normal hearing children attending mainstream school. Int J Pediatr Otorhinolaryngol..

[47] Zhang Z.G. (2023). The changes and characteristic of mapping after cochlear implantation. Chin J Urban Rural Enterp..

