# Supplementary material for: Correlation between free-field cortical auditory evoked potentials and behavioral audiometry thresholds in pediatric patients with hearing aids or cochlear implants
Source: Braz J Otorhinolaryngol. 2026 Nov 4;93(1):101922. doi: 10.1016/j.bjorl.2026.101922 (PMC13756624; doi:10.1016/j.bjorl.2026.101922)
Supplement: Supplementary file 1 [file mmc1.docx]

BJORL-D-26-00068_Supplementary Material

**Supplemental Figure 1** Relationship between free-field tone-burst CAEP P1 thresholds and free-field behavioral audiometry thresholds at 0.5 (A), 1 (B), 2 (C), and 4 kHz (D) in children with hearing aids. FF, Free Field; CAEP, Cortical Auditory Evoked Potential; HL, Hearing Level.

**Supplemental Figure 2** Relationship between free-field tone-burst CAEP P1 thresholds and free-field behavioral audiometry thresholds at 0.5 (A), 1 (B), 2 (C), and 4 kHz (D) in children with cochlear implants. FF, Free Field; CAEP, Cortical Auditory Evoked Potential; HL, Hearing Level.
